# Symptoms of diabetes distress, depression, and anxiety in people with type 2 diabetes: identifying central and bridge symptoms using network analysis

**DOI:** 10.1192/j.eurpsy.2022.736

**Published:** 2022-09-01

**Authors:** A. Mcinerney, N. Lindekilde, A. Nouwen, N. Schmitz, S. Deschenes

**Affiliations:** 1 University College Dublin, Psychology, Dublin, Ireland; 2 University of Southern Denmark, Psychology, Odense, Denmark; 3 Middlesex University, Psychology, London, United Kingdom; 4 University of Tübingen, Medicine, Tubingen, Germany

**Keywords:** Network Analysis, comorbidity, diabetes, diabetes-distress

## Abstract

**Introduction:**

People with diabetes are vulnerable to diabetes-related distress and are more likely to experience depressive and anxiety symptoms than the general population. Diabetes distress, depressive, and anxiety symptoms also tend to commonly co-occur.

**Objectives:**

This study aimed to apply network analysis to explore the associations between diabetes distress, depressive, and anxiety symptoms in a cohort of adults with type 2 diabetes.

**Methods:**

Data were from the baseline (2011) assessment of the Evaluation of Diabetes Insulin Treatment (EDIT) study (*N* = 1,796; 49% female; mean age = 60, *SD* = 8) from Quebec, Canada. A first network using the 17 items of the diabetes distress scale (DDS-17) was estimated. A second network was estimated using the 17 items of the DDS-17, the 9 depressive items of the PHQ-9, and the 7 anxiety items of the GAD-7. Symptom centrality, network stability, and bridge symptoms were examined.

**Results:**

Regimen-related and physician-related distress symptoms were amongst the most central (highly connected) in the diabetes distress network. *Worrying too much* (anxiety), *Not feeling motivated to keep up diabetes self-management* (diabetes distress), and *Feeling like a failure* (depression) were the most central symptoms in the combined network. *Feeling like a failure* (depression) was highly connected to diabetes distress symptoms, representing a potential bridge between diabetes distress and depression.

**Conclusions:**

Identifying central and bridge symptoms may provide new insights into diabetes distress, depressive, and anxiety symptom maintenance and comorbidity in people with type 2 diabetes.

**Disclosure:**

No significant relationships.

